# The evolution of precision oncology: The ongoing impact of the Drug Rediscovery Protocol (DRUP)

**DOI:** 10.2340/1651-226X.2024.34885

**Published:** 2024-05-23

**Authors:** Soemeya F. Haj Mohammad, Hans J.L. Timmer, Laurien J. Zeverijn, Birgit S. Geurts, Ilse A.C. Spiekman, Karlijn Verkerk, Florentine A.J. Verbeek, Henk M.W. Verheul, Emile E. Voest, Hans Gelderblom

**Affiliations:** a Department of Medical Oncology, Leiden University Medical Center, Leiden, the Netherlands; b Department of Molecular Oncology & Immunology, Netherlands Cancer Institute, Amsterdam, the Netherlands; c Department of Medical Oncology, Erasmus MC Cancer Institute, Rotterdam, the Netherlands

**Keywords:** Targeted therapy, immunotherapy, whole-genome sequencing, precision medicine

## Abstract

**Background and purpose:**

The Drug Rediscovery Protocol (DRUP) is a Dutch, pan-cancer, nonrandomized clinical trial that aims to investigate the efficacy and safety of targeted and immunotherapies outside their registered indication in patients with advanced or metastatic cancer.

**Patients:**

Patients with advanced or metastatic cancer are eligible when there are no standard of care treatment options left and the tumor possesses a molecular genomic variant for which commercially available anticancer treatment is accessible off-label in DRUP. Clinical benefit is the study’s primary endpoint, characterized by a confirmed objective response or stable disease after at least 16 weeks of treatment.

**Results:**

More than 2,500 patients have undergone evaluation, of which over 1,500 have started treatment in DRUP. The overall clinical benefit rate (CBR) remains 33%. The nivolumab cohort for patients with microsatellite instable metastatic tumors proved highly successful with a CBR of 63%, while palbociclib or ribociclib in patients with tumors harboring CDK4/6 pathway alterations showed limited efficacy, with a CBR of 15%. The formation of two European initiatives (PCM4EU and PRIME-ROSE) strives to accelerate implementation and enhance data collection to broaden equitable access to anticancer treatments and gather more evidence.

**Conclusion:**

DRUP persists in improving patients access to off-label targeted or immunotherapy in the Netherlands and beyond. The expansion of DRUP-like clinical trials across Europe provides countless opportunities for broadening the horizon of precision oncology.

## Introduction

With the rapidly evolving field of precision oncology, the Dutch Center for Personalized Cancer Treatment (CPCT) was established a decade ago to construct an integrated infrastructure for the collection of molecular genetics data linked to clinical outcome. This facilitated the creation of a database with information on patients with metastatic cancer to enhance the landscape of predictive and prognostic biomarkers in oncology [1]. By building this infrastructure, a network of 49 collaborating hospitals throughout the Netherlands was inaugurated. Concurrently, the nonprofit organization Hartwig Medical Foundation was instituted with the purpose of conducting whole-genome sequencing (WGS) and maintaining the database [2]. Priestley et al. conducted a large WGS analysis on this database, containing 2,520 genomic tumor landscapes [3]. They concluded that 62% of patients harbored at least one actionable target, for half of which an approved anticancer treatment was available. However, in 13% of patients, the possible treatment was outside its registered indication, suggesting that these patients would not have access to potentially beneficial treatment. To overcome this unmet need, the Drug Rediscovery Protocol (DRUP) was initiated, to provide patients the possibility of treatment with off-label therapies, with the intent to focus on broader implementation of already existing drugs, rather than developing novel treatments. This investigator-initiated study aims to investigate the efficacy and safety of commercially available anticancer treatments in patients with advanced or metastatic cancer that have no more standard of care treatment options left and provide patients access to these medications based on their tumors’ molecular profile [4]. Here we present an overview of DRUP, illuminating implications for the future.

## Methods

### Study design

**Figure 1:**
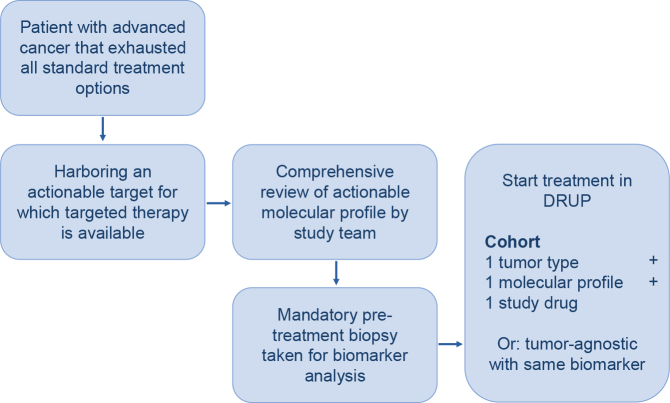
Study design.

DRUP is a Dutch, ongoing, multicenter, nonrandomized, prospective, umbrella, and basket trial in which patients receive off-label treatment with commercially available targeted therapy or immunotherapy based on potentially actionable molecular alterations in their tumor. Parallel cohorts are designed for patient enrolment, characterized by tumor type, molecular alteration and study treatment, as well as tumor-agnostic cohorts (Figure 1) [4].

Approval for DRUP (NCT02925234) was granted by the Medical Ethics Committee at the Netherlands Cancer Institute in Amsterdam, the Netherlands, following the guidelines for Good Clinical Practice and ethical principles for medical research from the Declaration of Helsinki. The protocol and study design have been published earlier in more detail [4].

### Study population

Patients are eligible for the trial if they have a progressive advanced or metastatic solid tumor, multiple myeloma, or non-Hodgkin lymphoma and have no standard treatment options left. A potentially actionable genomic variant has to be identified as part of routine molecular diagnostics. Furthermore, patients need to be adults ≥18 years of age, have an adequate performance score and organ function, and have measurable disease according to the RECIST 1.1, RANO, IMWG, or Lugano, for solid tumors, high-grade gliomas, multiple myeloma, or lymphoma, respectively, which is necessary for radiological response evaluations [5–8].

### Matching treatment and assessments

The study team assesses each case submission by aiming to match a specific molecular alteration with an available therapy in DRUP. For each therapy, potentially actionable alterations were predefined according to literature [9]. A more detailed description of the matching rules has been published previously [4]. After a successful match, patients are enrolled in the study and undergo screening according to drug-specific inclusion criteria. Radiological response evaluations are performed at baseline and every 8 or 9 weeks after start of treatment, depending on the study treatment. The primary endpoints are clinical benefit (CB) and safety. CB is defined as confirmed complete or partial response or stable disease after 16 weeks of treatment. Safety is measured by the frequency of grade ≥3 treatment-related adverse events following the Common Terminology Criteria for Adverse Events (CTCAE) version 4.03. Objective response rate (ORR) is defined as the percentage of patients with complete or partial response.

### Pretreatment biopsies

Before treatment is initiated, a mandatory fresh frozen biopsy specimen is obtained, together with a blood sample that is solely used to assess a patient’s germline DNA, and sent to Hartwig Medical Foundation where WGS and RNA sequencing is performed if the tumor biopsy consists of a certain tumor-cell percentage [10]. Patients with primary brain tumors are exempt from this procedure, similar to patients for whom WGS was performed in a nonclinical trial setting prior to study enrolment, without receiving any anticancer treatment in between, as well as patients who underwent an allogenic stem cell transplantation in the past, due to the inability to perform accurate WGS because of the mismatch between the biopsy specimen and blood sample.

### Statistical analysis

**Figure 2:**
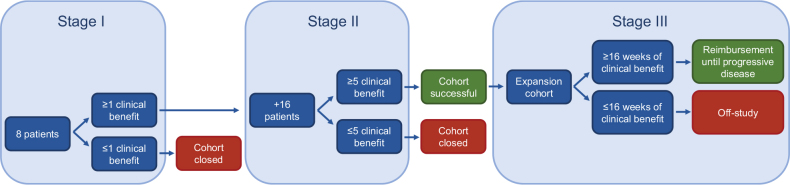
Cohort design.

A Simon-like two-stage design is used to monitor cohorts (Figure 2) [11] Stage 1 consists of eight patients, of which at least one patient needs to show CB for the cohort to stay open and extend to stage 2, where 16 additional patients are added. If then five or more patients meet the criteria for CB, the cohort is considered successful and gives opportunities for more extensive investigation. An 85% power and a one-sided alpha error rate of 7.8% is used to reject the null hypothesis of a 10% clinical benefit rate (CBR) if the true CBR is ≥30%, as described in earlier publications [12, 13].

## Results

Since the initiation of DRUP in 2016, as of November 2023, over 2,500 patients have been submitted to the study team for potential study participation. More than 1,500 of these patients started treatment with one of the 36 available targeted- or immunotherapies in DRUP, provided by 14 different pharmaceutical companies, at one of the 35 affiliated hospitals across the country. An interim analysis of the first 500 included patients showed a CBR of 33% with similar results between rare and nonrare cancers [14].

An example of a successful stage 2 cohort is the tumor-agnostic nivolumab cohort for patients with tumors that harbor microsatellite instability (MSI), with a CBR of 63% [4]. Based on these results, a third stage cohort was opened to validate the findings from this second stage cohort in a broader population (Figure 2). This ‘expansion’ cohort showed promising results with a CBR of 62% and an ORR of 45% [15]. While the therapies in the first and second stage cohorts are provided free of charge by the pharmaceutical companies, a personalized reimbursement model was created for third stage cohorts to facilitate risk sharing between the pharmaceutical companies and healthcare insurers. In this model, treatment coverage is transferred to the healthcare insurers after a patient shows CB for at least 16 weeks [16]. The Dutch National Health Care Institute analyzed the positive preliminary stage 3 findings and provided a positive recommendation for the reimbursement of nivolumab for patients with pretreated advanced MSI tumors, regardless of tumor type. Consequently, these patients now have access to nivolumab outside its registered label since July 1st 2022 in the Netherlands [17]. This paves the way for more new reimbursed indications based on data from DRUP. Another promising second stage cohort is the cohort for BRCA1 and BRCA2 mutated tumors treated with olaparib. It was shown that 58% of the treated patients had CB with an ORR of 29%, therefore laying the groundwork for a possible new stage 3 cohort in the future [18]. Furthermore, patients with MET-mutated non-small cell lung cancer (NSCLC) treated with crizotinib responded extraordinarily well, with a CBR of 71% and an objective response in 62% of patients [19].

Nonetheless, it is important to note that not only successful DRUP cohorts are being published, as exemplified in a recent article by Zeverijn et al. [20]. The article reported the results of 139 patients with various tumor types, either enrolled in DRUP or the Australian Cancer Molecular Screening and Therapeutic (MoST) trial, that were treated with one of the CDK4/6 inhibitors palbociclib or ribociclib as monotherapy, based on complete loss of CDKN2A or SMARCA4 or amplifications of CDK4, CDK6, CCND1, CCND2, or CCND3. These patients exhibited limited clinical efficacy with a CBR of 15% and the absence of any objective response. Based on these findings accrual for these agents was terminated and it was concluded that palbociclib or ribociclib administered as monotherapy is not recommended.

With 218 cohorts in DRUP open for accrual at this point, it has become evident that certain combinations of molecular profiles, tumor types and treatments are very rare. Over the years, 44 cohorts have progressed to stage 2, while the remaining cohorts persist in stage 1, resulting in a median of 2 patients per cohort. While the primary goal is to provide access to potentially effective medicine to as many patients as possible, it is equally crucial to gather evidence for further implementation of these treatments. In 2021, a Memorandum of Understanding was signed between DRUP and precision oncology trials in the Nordic countries (Denmark, Norway, Sweden, and Finland), which based their protocols on the DRUP protocol to ensure alignment in trial design and endpoints, also referred to as DRUP-like clinical trials. This alignment grants the ability to share data and accelerate evidence gathering [21]. Building further on this Memorandum, two European projects were launched in 2023: Precision Cancer Medicine for all EU Citizens (PCM4EU), funded by the EU4Health program as part of Europe’s Beating Cancer Plan (grant: 101079984), and Precision Cancer Medicine Repurposing System Using Pragmatic Clinical Trial (PRIME-ROSE), funded under the Horizon Europe program (grant: 101104269), aiming to broader implement DRUP-like clinical trials across the European Union. This network of independent investigator-initiated DRUP-like clinical trials focusses on data sharing to ensure equitable access to effective anticancer treatment within Europe [22].

## Discussion

Since 2016, DRUP has been extending access to off-label anticancer treatments for patients with advanced or metastatic cancer. This article exemplifies the ongoing impact of this precision oncology trial. Notably, the outcomes of DRUP have led to the reimbursement of nivolumab for all patients in the Netherlands with pretreated advanced tumors that harbor MSI, highlighting the potential opportunities an investigator-initiated study can provide [4, 17]. Concurrently, addressing the limited therapeutic efficacy of palbociclib or ribociclib as monotherapy for patients with alterations in the cyclin D-CDK4/6 pathway, underscores the importance of reporting negative evidence to prevent futile treatments in the future [20].

The ongoing CBR of 33% observed in DRUP demonstrates that matched targeted anticancer therapies benefit a considerable number of patients [14]. However, the remaining two-thirds of patients do not show CB to this therapeutic approach, with drug resistance standing out as a primary contributor to the unresponsiveness to treatment. Despite the extensive research on the field of resistance, intrinsic as well as acquired, conquering this hurdle continues to be challenging [23]. A better understanding of the underlying mechanisms for resistance to targeted monotherapy provides opportunities for optimized treatment possibilities, including combination therapy, together with improved patient selection, even within the framework of DRUP, underlining the importance of broad implementation of genomic testing.

Some limitations warrant consideration in the context of this study. Primarily, the diversity of the study population might challenge data interpretation. Additionally, the absence of a comparison group could complicate the interpretation of the treatment efficacy. Nevertheless, it is essential to acknowledge the ethical objections of withholding patients potentially effective treatment while they have no therapeutic options left. To overcome these challenges, novel methodologies have been suggested, incorporating the utilization of real-world data as a control mechanism [24]. This approach is being investigated within the framework of the European project PRIME-ROSE.

In conclusion, DRUP continues to provide patients with advanced or metastatic disease access to off-label targeted or immunotherapy based on their genetic tumor profile. The establishment of European collaborations of DRUP-like clinical trials holds promise for expanding the scope of precision oncology.

## Data Availability

All data described in this study are freely available for academic use and can be obtained through a request to the corresponding author by email.
